# Patient Reported and Clinical Outcomes after High-Dose Chemotherapy and Autologous Stem Cell Transplantation in Primary Central Nervous System Lymphoma

**DOI:** 10.3390/cancers15030669

**Published:** 2023-01-21

**Authors:** Sina A. Beer, Stefan Wirths, Wichard Vogel, Ghazaleh Tabatabai, Ulrike Ernemann, David A. Merle, Wolfgang Bethge, Robert Möhle, Claudia Lengerke

**Affiliations:** 1 Department of Internal Medicine II, Hematology, Oncology, Clinical Immunology and Rheumatology, University Hospital Tübingen, 72076 Tübingen, Germany; 2 Department of Neurology and Interdisciplinary Neuro Oncology, Hertie Institute for Clinial Brain Research, University Hospital Tübingen, 72076 Tübingen, Germany; 3 Center for Neuro-Oncology, Comprehensive Cancer Center Tübingen-Stuttgart, 72076 Tübingen, Germany; 4 Department of Neuroradiology, University Hospital Tübingen, 72076 Tübingen, Germany; 5 Department of Ophthalmology, University Hospital Tübingen, 72076 Tübingen, Germany

**Keywords:** autologous hematopoietic stem cell transplantation, non-Hodgkin lymphoma, PCNSL, patient-reported outcome, quality of life

## Abstract

**Simple Summary:**

Primary central nervous system lymphomas are rare, but the incidence in the elderly population increases constantly. Consequently, more and more elderly patients are treated with high-dose chemotherapy followed by autologous stem cell transplantation (HDC/ASCT). However, data on the recovery after this demanding therapy are scarce, especially concerning quality of life (QoL)-focused patient-reported outcome parameters. Seeing even better QoL results in the elderly compared to the younger population after HDC/ASCT this single-center analysis challenges the assumption of an insufficient recovery by seeing even better QoL results in the elderly compared to the younger population after HDC/ASCT. Moreover, no significant age-dependent differences were observed regarding overall and progression free survival as well as ECOG performance status and mini-mental state examination. Together, our data indicate that HDC/ASCT is an effective therapy with respect to disease control and global health status.

**Abstract:**

Primary central nervous system lymphomas (PCNSL) are rare and associated with an adverse prognosis. High-dose chemotherapy followed by autologous stem cell transplantation (HDC/ASCT) improves progression free (PFS) and overall survival (OS) but neurocognition, performance status and quality of life (QoL) in patient-reported outcome (PRO) after HDC/ASCT remains underexplored. Especially elderly patients may insufficiently recover from this demanding therapy. Therefore, this single-center analysis investigated all PCNSL patients who received HDC/ASCT at the University Hospital Tübingen from 2006–2021 (*n* = 40, median age 60.5 years) in a retrospective manner. The 2-year PFS/OS was 78.7%/77.3%, respectively, without significant differences between the tested age-groups (≤60 vs. >60 years, *p* = 0.531/*p* = 0.334). Higher Thiotepa dosage was an independent predictor for better OS (*p* = 0.018). Additionally, a one-time prospective, cross-sectional analysis after HDC/ASCT in the same cohort was performed (*n* = 31; median follow-up 45 months). Here, the median ECOG improved by HDC/ASCT from 1 to 0 and mini-mental state examinations revealed unimpaired neurocognitive functioning (median 28 pts.). PRO data collected by EORTC QLQ-C30 showed a good QoL in both age groups with an average global health status (GHS) of 68.82% (≤60y: 64.72%, >60y: 74.14%). Together, our data indicate that HDC/ASCT is an effective therapy with respect to disease control, overall health status and quality of life, irrespective of patient age.

## 1. Introduction

Primary central nervous system lymphomas (PCNSL) belong to the group of aggressive extra nodal non-Hodgkin lymphomas [1] and mainly classify as diffuse large B-cell lymphomas (DLBCL) [2,3]. Compared to systemic lymphomas, PCNSLs present with a distinct biology with disparate molecular features [1]. This may be due to their emergence in the central nervous system (CNS) and its immune-privileged status. From an epidemiological perspective, PCNSLs are rare cancers with a median age at diagnosis of 65 years (y) [4] and an increasing incidence with age [5,6]. In line with this, today, up to 20% of all PCNSL patients are older than 80 years [7].

PCNSLs are sensitive to chemo- as well as radiotherapy. Whole brain irradiation therapy (WBRT) and intensified systemic treatment regimens have been shown to induce long term remissions or even cure [8]. While highly effective, WBRT was however reported to associate with severe neurotoxicity accompanied by reduced quality of life (QoL) [9] and is therefore no longer recommended as 1st line treatment. One first radiation-free therapy regime was tested in the “Bonn protocol” using systemic therapy in combination with intraventricular therapy [10], showing long-term disease control in a subset of patients [11]. Further, the efficacy of high dose chemotherapy (HDC) and autologous stem cell transplantation (ASCT) was tested [12] and led to a 70% 7-year overall survival (OS) in a phase III clinical trial [13]. Especially in younger patients (<65y), HDC/ASCT demonstrated an excellent response rate of 80 to 96% and a low mortality of 0 to 12% [14]. In addition, HDC/ASCT showed significant lower neurotoxicity when compared to WBRT (49% vs. 26%) [15]. However, patients over 60 years are more vulnerable to side effects and often receive dose adjusted protocols [16]. They are at higher risk for developing neurotoxicity and show a lower 5-year OS of 30% after HDC/ASCT [17], with a yet underexplored and therefore questionable QoL benefit. Generally, only a few studies focusing on the QoL of PCNSL patients were published to date, but a trend towards a QoL improvement after induction treatment was observed [15,18].

Therefore, this study investigated the outcome of PCNSL patients after HDC/ASCT with special attention to elderly patients (>60 years) and their potential benefits concerning neurocognition, performance status (PS) and quality of life (QoL) in patient-reported outcome (PRO).

## 2. Patients and Methods

### 2.1. Patient Cohort and Clinical Data Sources

This single-center analysis included all patients diagnosed with PCNSL, who underwent HDC/ASCT in any line between 2006 to 2021 at the University Hospital Tübingen. The local Institutional Review Board approved this study (no. 376/2022BO2). Data collection was performed using the following databases: the Comprehensive Cancer Center Tübingen (CCC), the Koordobas System and the German Register for Stem Cell Transplantation (DRST). PCNSL patients with an age of 18 years or older were identified by filtering for the diagnosis PCNSL (ICD-10-GM-2022: C83.3) in combination with HDC/ASCT treatment. Systemic lymphoma patients with CNS manifestation and immunocompromised patients were excluded. Electronic medical records were reviewed for the following information: demographic data, initial disease status, laboratory values, treatment regimens including induction and consolidation therapy, amount of infused CD34+ stem-cells, therapy-induced complications and treatment-related mortality (TRM) until day 100, time to engraftment and long-term cytopenia. Relapses, secondary malignancies, long-term complications, PFS and OS were assessed. The study population was stratified by age (groups: ≤60 vs. >60y).

### 2.2. Assessment of Radiologic Responses

Radiological response was analyzed in cMRI scans prior to and 3, 12 and 24 months after ASCT. MRI scans were analyzed utilizing the International PCNSL Collaborative Group (IPCG) Consensus Guidelines and were rated as complete response (CR), unconfirmed CR (CRu), partial response (PR), stable disease (SD) or progress (39).

### 2.3. Progression Free Survival and Overall Survival

PFS was calculated as the time from transplantation to date of progression, death or last follow-up, whichever came first. OS was defined as the time from date of transplantation to death of any cause.

### 2.4. Definition of Engraftment and Cytopenias

Neutrophil engraftment was defined as the first of three consecutive days with an absolute neutrophil count of at least 0.5 × 10^9^/L. A platelet count exceeding 20 × 10^9^/L without platelet transfusion was classified as engrafted. Delayed engraftment was defined as not fulfilling the criteria mentioned above at day 28 after transplantation [19]. During follow-up care, a leukocyte count of ≥4 × 10^9^/L and a thrombocyte count of ≥150 × 10^9^/L were defined as normal values [20].

### 2.5. Performance Status, Patient-Reported and Neurocognitive Outcome

Performance status (PS), patient-reported outcome (PRO) and MMSE were collected using one-time and cross-sectional methods at the latest visit. The performance status (PS) and MMSE were used as clinical anchors in follow-up examinations. Follow-up intervals were calculated in months from ASCT to the last visit of the patient. PS was stratified according to the ECOG scale from 0–5 at the time of diagnosis and last visit. MMSE evaluation was used as neurocognitive bedside test and was also conducted at the last follow-up visit, if necessary, with translation by family members. MMSE has a maximum score of 30 points and lower scores indicate cognitive or memory deficits [21]. Additionally, we surveyed PRO using the EORTC QLQ-C30 V.3 questionnaire designed by the European Organization for Research and Treatment of Cancer [22]. Based on item 29 and 30 of EORTC QLQ-C30, we assessed the patient’s global health status (GHS). PRO was a pre-specified primary endpoint. Patients were encouraged to complete the questionnaire during their stay at the clinic. Alternatively, the patients returned the questionnaire by mail.

### 2.6. Statistical Methods

Statistical analyses were performed using the Software Excel (Microsoft Office Professional Plus 2019) and IBM^®^ SPSS Statistics 28. The patient characteristics were expressed as frequencies or categorical variables. Categorical data were compared via a chi-square test or Fisher’s exact test. Survival times were calculated using the Kaplan–Meier method and specified via a log-rank test. Cox’s regression analysis was used to estimate the effect on OS of various variables (age, sex, radiologic response, TT dosage). We considered competing risks to estimate the cumulative incidence regarding toxicity and treatment-related mortality (TRM) in the cohort. We used descriptive statistics to summarize PRO data, MMSE and ECOG. We did not add values of EORTC QLQ-C30 questionnaires that were missing. PRO values were calculated based on the official scoring manual [23]. The association between parameters was assessed by using Pearson’s correlation or Phi coefficient for dichotomous variables. Moreover, statistical analyses on the prognostic impact of all parameters mentioned above were performed in both age groups (≤60 vs. >60y) utilizing t- or nonparametric tests depending on distribution and data type. Due to the limited sample size non-parametric tests were used in most instances. A *p*-value of <0.05 was considered statistically significant.

## 3. Results

### 3.1. Patient Cohort and Therapy Regimens

ASCT was performed in 40 PCNSL patients (17 female, 23 male) with a median age of 60.5 years (range 24–80, 27.5% > 65y) at ASCT. *N* = 30 (75%) received 1st line HDC/ASCT and *n* = 28 (70%) patients were treated outside of clinical trials. All patients had a histologically confirmed DLBCL. A total of 75% demonstrated an ECOG of 0 or 1, and 25% had an ECOG of 2 or 3 at time of initial diagnosis. The median time from first diagnosis to ASCT was 4 months (range 2–91). Patient and therapy characteristics in the whole cohort are outlined in Table 1. All patients were treated with an MTX-containing induction chemotherapy, including Rituximab. A total of 15% (*n* = 6) had impaired renal function and were consequentially treated with a reduced MTX-dosage. The conditioning regime contained Thiotepa (TT) for all patients but differed in dosage: 26 patients (65%) received 4 × 5 mg/kg and 14 patients (35%) 2 × 5 mg/kg. A total of 28 patients (70%) received TT in combination with Carmustin (BCNU) and 12 patients (30%) received TT-Busulfan.

Patients who received 2nd (*n* = 8) or 3rd line (*n* = 2) HDC/ASCT had the following therapy regimens upfront: MTX/Ifosfamid (*n* = 2), R-DeVIC (*n* = 3), R-MTX (*n* = 4) or WBRT (*n* = 1). Patients experiencing relapse after HDC/ASCT were treated with WBRT (n = 4), R-Temozolomid (*n* = 1), Ibrutinib (*n* = 1) or R-DeVIC (*n* = 1), respectively.

**Figure 1:**
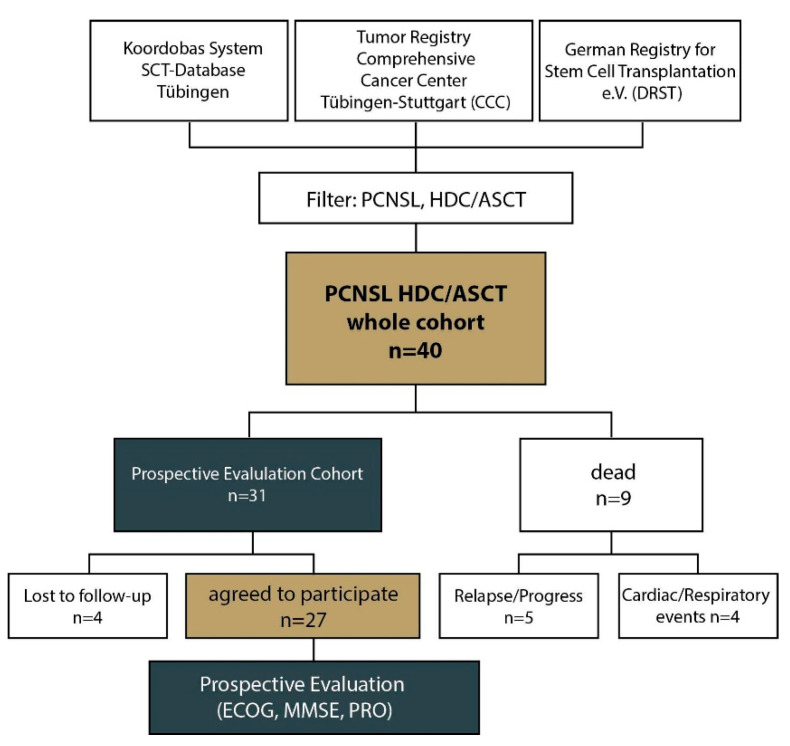
Study workflow. By screening the CCC, the Koordobas and the DRST databases using the diagnosis primary central nervous system lymphoma (PCNSL) in conjunction with high-dose chemotherapy followed by autologous stem cell transplantation (HDC/ASCT) we identified *n* = 40 PCNSL patients aged 18 years or older. Among those, 31 were alive and 9 had died. The 31 living patients were contacted for prospective evaluation with performance status (using the ECOG scale), MMSE and PRO. Four patients were lost to follow-up. Abbreviations are as follows: CCC—Comprehensive Cancer Center Tübingen–Stuttgart; DRST—German Register for Stem Cell Transplantation; MMSE—mini-mental state examination; PRO—patient-reported outcome.

A one-time prospective, cross-sectional analysis of PS, MMSE and PRO after HDC/ASCT was performed in 31 patients (Figure 1). Patient characteristics in the prospective evaluation cohort are outlined in Table 2.

### 3.2. Clinical Outcome and Radiologic Responses

**Figure 2:**
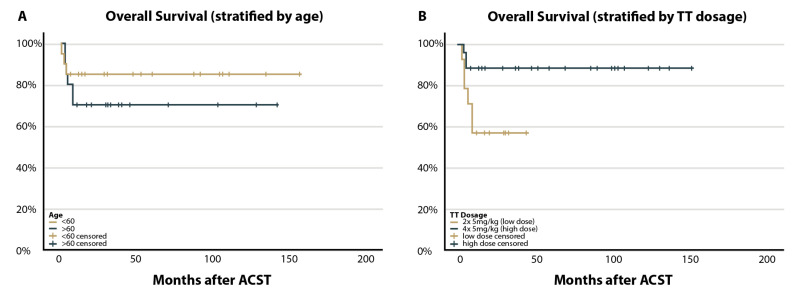
Overall survival (OS). X-axis: time of initial diagnosis until today. Y-axis: OS in %. Kaplan–Meier overall survival curve of PCNSL patients treated with high-dose chemotherapy followed by autologous stem cell transplantation (HDC/ASCT). (**A**): Stratified by age (≤60 years vs. >60 years) no significant differences in OS were observed (*p* = 0.334). (**B**): Stratified by TT dosage (group 4 × 5 vs. 2 × 5 mg/kg) a significant difference is seen in OS (*p* ≤ 0.001).

PFS and OS, mortality rate and radiologic responses were retrospectively evaluated in the whole cohort. The 2-year PFS and OS were 78.7% and 77.3% (r = 0.066), respectively. An average OS of 122.4 months (95% CI 102.5–142.3) was observed. Stratified by age, no significant differences in PFS and OS were observed (*p* = 0.531 and *p* = 0.334), but the younger group demonstrated a trend towards better OS (Figure 2A). Seven patients (17.5%) relapsed after upfront HDC/ASCT within the first 6 months. Treatment related mortality (TRM) at day 100 was 5% (*n* = 2). Seven patients died, four due to relapse or progression and three due to respiratory complications. Two other patients died during the longer follow-up, one due to progress and one due to cardiac decompensation. A trend towards a higher risk for early deaths after HDC/ASCT in the elderly cohort can be seen (Figure 2A). Two of the relapsed patients showed longer-term survival: one treated with WBRT (follow-up month 130) and one with R-Temozolomid (follow-up month 71).

Interestingly, analyses stratified by TT dosage (group 4 × 5 vs. 2 × 5 mg/kg) revealed a significant difference in OS (*p* ≤ 0.001, Figure 2B). TT dosage correlated positively with OS (r = 0.478). In a multivariate analysis, TT dosage was an independent predictor for OS (*p* = 0.018).

Radiologic responses were retrospectively evaluated in the whole cohort. In cMRI scans following induction treatment, twelve patients (30%) showed CR or CRu, twenty-four (60%) had a PR, four (10%) demonstrated a mixed response and two (5%) showed progress. After HDC/ASCT, a CR or PR was observed in 24 (60%) and 12 (30%) patients, respectively, corresponding to a 90% overall radiological response rate. Comparing the group with CR to the group with PR after ASCT, there was a trend towards improved OS with CR (*p* = 0.071). No significant difference in OS was seen between PR and CR before ASCT (*p* = 0.414) and no correlation between radiologic response and TT dosage was found.

### 3.3. ASCT Parameters

ASCT parameters were retrospectively analyzed in the whole cohort. The median number of reinfused CD34+ hematopoietic stem cells was 4.015 × 10^6^/kg (range 0.51–45.34). The median granulocyte- and platelet take was observed at days 10 (range 8–210) and 12 (range 6–180), respectively. Delayed granulocyte or platelet recovery was observed in one and six patients, respectively. Interestingly, 19 patients (49%) failed to regenerate hematopoiesis to normal levels in long-term follow-up [20]. Sixteen patients (40%) showed persisting thrombocytopenia, fourteen of these in a range from 100–150 × 10^3^/µL, one patient required thrombocyte transfusions until last follow-up and one was diagnosed with immune thrombocytopenia and received TPO-agonists. Delayed engraftment did not significantly correlate with transplanted CD34+ cell counts (*p* = 0.177) or TT dose (*p* = 0.330) and did not affect PFS or OS (*p* = 0.633 or *p* = 0.379).

Mucositis, commonly accompanied by nausea, was the most common non-hematologic grade 3 or 4 toxicity until day 30 after ASCT and occurred in 21 patients (52.5%). A total 25 patients (62.5%) experienced fever in aplasia, but there was no detectable association with transplanted CD34+ cell counts (*p* = 0.492). Other observed acute complications were catheter infections with bacteriaemia (*n* = 8, 20%), catheter vein thrombosis (*n* = 3, 7.5%), clostridium difficile infections (*n* = 5, 12.5%) or cerebral ischemia (*n* = 3, 7.5%).

### 3.4. Prospective Analysis of Surviving Patients with PCNSL and HDC/ASCT

**Figure 3:**
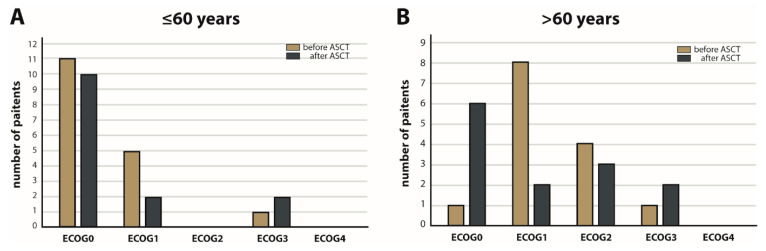
ECOG performance status (PS) before and after high-dose chemotherapy followed by autologous stem cell transplantation (HDC/ASCT). X-axis: ECOG PS grade 0–4. Y-axis: number of patients. (**A**): Group ≤ 60 years (*p* = 0.414). (**B**): Group > 60 years (*p* = 0.449).

The surviving cohort treated with HDC/ASCT were prospectively analyzed in a cross-sectional manner (*n* = 31, Figure 1) with respect to PS, neurocognition and patient-reported outcome (PRO), including QoL. A median ECOG of 1 (SEM 0.885) before and 0 after HDC/ASCT (SEM 1.145) was noted, caused by a shift to ECOG 0 with 40% (after ASCT) vs. 30% (before ASCT). The PS before HDC/ASCT correlated positively with the PS after ASCT (*p* = 0.003). The positive effect of HDC/ASCT on PS in the surviving cohort did not reach significance considering the whole cohort (*p* = 0.790) or subgroup analyses (patients ≤ 60y, *p* = 0.414, Figure 3A; >60y, *p* = 0.449, Figure 3B).

A total of 27 PCNSL patients were available for a posttreatment neuropsychological evaluation. Unspecific persistent neurocognitive impairments (e.g., memory problems, dizziness, walking unsteadiness) were found in 51.9% of patients, of whom 41.9% still received levetiracetam as part of their long-term medication. A total of 26 patients completed a MMSE follow-up evaluation with 28 points in median. Only five patients qualified for mild (*n* = 4) or moderate dementia (*n* = 1). MMSE results were independent of age (≤60 vs. >60y, *p* = 0.600) or sex (women vs. men, *p* = 0.643).

**Figure 4:**
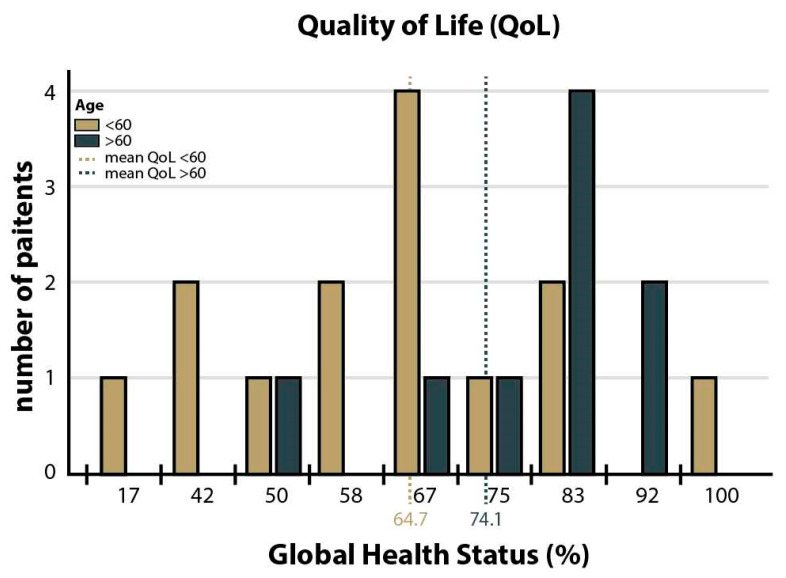
Global health status (GHS) stratified by age. X-axis: GHS in %. Y-axis: number of patients. The global health status (GHS) of PCNSL patients after high-dose chemotherapy followed by autologous stem cell transplantation (HDC/ASCT) in the EORTC QLQ-C30 questionnaire. An averaged GHS of 68.82% (range 17–100%) was documented. Stratified by age, 64.72% (≤60 years) and 74.14% (>60 years), no significant difference is seen (*p* = 0.241).

Prospective patient reported outcome analysis could be performed in 23 of 31 patients who returned a completed EORTC QLQ-C30 questionnaire. This corresponds to a 74.2% reply rate in the survival cohort. The descriptive analysis of the QoL data revealed an averaged global health status (GHS) of 68.82% (range 17–100%). The GHS was by trend higher in elderly as compared to younger patients (>60y: 74.14% vs. ≤60y: 64.72%, *p* = 0.241, Figure 4) but not influenced by ECOG group (0–1 vs. ≥2, *p* = 0.843) or radiological response (CR vs. PR, *p* = 0.884). The five functional subscales in the EORTC QLQ-C30 revealed normal physical and emotional functioning with 76.7% and 82.2%, respectively. Cognitive, role and social functioning were impaired with 64.5%, 63.1% and 49.3%, respectively. Regarding the symptom scales, the predominant issue for patients was fatigue (32.4%), followed by insomnia (13.9%) and pain (11.1%). Women did not state more symptoms in comparison to men (*p* = 0.812).

## 4. Discussion

Our single-center study suggests that HDC/ASCT is a safe and effective therapy in PNCSL, regardless of age. The survival benefit of elderly patients in our cohort stands in contrast to recently published results of a multi-center review, which postulates worse outcomes in patients over 60 years [17]. An interesting observation was the positive correlation between Thiotepa (TT) dosage and overall survival, which was independent of age. In the group > 60y, 40% received 4 × 5 mg/kg (high dose) TT as part of the consolidation despite their age. Subgroup analysis identified a survival benefit for this population. However, notably, the oldest patient with high-dose TT was 65 years old. Overall, our results might indicate that treatment intensification, if tolerable, enhances survival rates in PCNSL patients and may also be applicable also in elderly. To date, randomized trials assessing different TT dosages are missing and dose stratification is so far only directed by age [24,25]. Given the reported higher risk of infective complications in the group > 60y when applying intensive therapies [26], a TT dose reduction is usually performed in patients over 70 years [26,27]. Considering our small sample size and the retrospective character of our study, these results require further validation in prospective trials.

In the second part of our study, we prospectively measured bedside neuropsychological parameters and QoL as primary endpoints in addition to classical transplantation outcomes. Interestingly, PCNSL patients treated with HDC/ASCT showed nearly similar global health status (GHS) scores compared to the general German population (68.82% vs. 70.8%) [28]. The occurrence of unspecific cognitive impairments in 51.9% of patients was comparable to published data [29] but cognitive functioning as assessed by PRO was surprisingly unaffected with a score of 64.5%. Another study found this parameter to be as low as 28.8% [30]. MMSE results with a median of 28 points also plead for no relevant cognitive decline in the majority of PCNSL patients. However, MMSE data should be interpreted carefully as it only displays a screening method for neurocognition. The general analysis of MMSE in elderly indicates a cut-off at 26 points for cognitive decline up to 93 years [31]. Intriguingly, patients of over 60 years showed a trend towards better GHS scores than the younger patients in follow-up care (74.14% vs. 64.72%, *p* = 0.241). Selection bias might influence these scores, since patients over 60 years were possibly more stringently selected prior to allocation to HDC/ASCT (e.g., for absence of comorbidities and performance status). However, similar results were observed in a phase II study investigating QoL after allogenic transplantation in elderly patients [32].

Notably, most of the scoring systems currently used in clinical routine lack proper risk stratification due to outdated age cut-offs [33], e.g., 50 years in MSKCC (Memorial Sloan–Kettering Cancer Center) and 60 years in IELSG (International Extranodal Lymphoma Study Group) score. An alternative score published in 2020 is the Taipai score with an age cut-off of 80 years [34]. The Taipai score could not be performed in our cohort because patients above 80 years were missing. Taken together, there is a lack of well applicable prognostic scores to perform adequate risk stratification in the aging population.

The age-independent, hardly affected results obtained in MMSE and PRO suggest a surprisingly low risk for long-term neurotoxicity after HDC/ASCT in our patient cohort. Unfortunately, most studies in this field are more than 10 years old and the influence of HDC/ASCT remains unclear to some extent. Looking at our results, one has to consider the median follow-up of 31 (overall cohort) and 45 months (prospective cohort). We did not sufficiently cover long-term-results because only a subgroup of patients completed 3 or more years of follow-up. In addition, the risks of this treatment are probably underestimated, because it lacks standardized neuropsychological testing in daily routines. Hence, in the present study pre-treatment neurological examinations, including MMSE, were not consistently performed at first diagnosis, and the MMSE starting point of the patients prior to disease development naturally remains obscure. MMSE has a rather low sensitivity for the evaluation of neurocognitive function [35], but the reasonable expenditure of time for patients makes it feasible in a daily routine. By contrast, the EORTC QLQ-30 for PRO assessment has a high sensitivity in testing QoL, but analysis and interpretation are extensive and complex. One other limiting factor in PRO research is that it requires a large degree of patients’ collaboration. As a result, the return rates of adequately answered questionnaires are often low. Further studies should verify whether optimized PRO assessments as well as neurocognitive and neurologic examinations are adequate to monitor the GHS of patients. Moreover, the dynamic nature of the construct QoL has to be methodically considered. Multiple assessments over time could be useful to reduce potential biases introduced by environmental influences. The implementation of electronic alternatives in a remote setting is a future goal and was recently begun in some pilot projects [36].

The assumption that particularly elderly patients do not sufficiently recover after the demanding therapy with HDC/ASCT is challenged by our data. Instead, our data support the concept that HDC/ASCT preserves and improves the health status and should also be considered in elderly PCNSL patients. Due to the aging population and the increasing incidence of PCNSL in the elderly, a growing number of patients will require HDC/ASCT or comparable treatments in the future. It is important to highlight the lack of information regarding long-term outcome, especially with respect to QoL in this cohort. In the changing landscape of new therapies (e.g., Bruton tyrosine kinase inhibitors, lenalidomide [37] and, potentially, CAR-T cells) adapted prognostic scores should be developed to better predict QoL and neurocognition before and after different treatments. Such scores could complement clinical outcome analyses, facilitate decision making and specifically evaluate the aging population.

## 5. Conclusions

In conclusion, we think that older age by itself should not be considered as absolute contraindication for HDC/ASCT as long the other eligibility criteria are met. Forcing multi-center trials to investigate concepts which maximize both the duration of response and QoL in PCNSL patients should by prioritized.

## Figures and Tables

**Table 1:** Patient characteristics in the whole cohort (*n* = 40).

Characteristic	Data
Gender	Female 42.5% (17/40), Male 58.5% (28/40)
Average age at diagnosis, years (range)	58 (24–77)
Average age at ASCT, years (range)	58 (24–80)
Age >60 years at ASCT	50% (20/40)
Deep brain structure	50% (20/40)
Histologic subtyp: DLBCL	100% (40/40)
Median ECOG before ASCT, grade	1
ECOG before ASCT, grade 0 or 1	75% (30/40)
Median follow-up, months (range)	31 (1–157)
Therapy in study	30% (12/40)
1st line HDC/ASCT	75% (30/40)
Induction treatment, Rituximab-containing	100% (40/40)
Consolidation treatment, TT-containing	100% (40/40)

**Table 2:** Patient characteristics in the prospective evaluation cohort (*n* = 31).

Characteristic	Data
Gender	Female 45.1% (14/31), Male 54.9 (16/31)
Average age at last follow up	63 (26–80)
Average age at diagnosis, years (range)	56 (24–77)
Average age at ASCT, years (range)	56 (24–77)
Age >60 years at ASCT	45.1% (14/31)
Deep brain structure	51.6% (16/31)
Histologic subtyp: DLBCL	100% (31/31)
Median ECOG before ASCT, grade	1
ECOG before ASCT, grade 0 or 1	80.6% (25/31)
Median follow-up, months (range)	45 (7–157)
Therapy in study	32.2% (10/31)

## Data Availability

The data presented in this study are available on request from the corresponding author. The data are not publicly available due to privacy.
